# Influence of Prior Beliefs on Perception in Early Psychosis: Effects of Illness Stage and Hierarchical Level of Belief

**DOI:** 10.1037/abn0000494

**Published:** 2020-08

**Authors:** Joost Haarsma, Franziska Knolle, Juliet D. Griffin, Hilde Taverne, Marius Mada, Ian M. Goodyer, The NSPN Consortium, Paul C. Fletcher, Graham K. Murray

**Affiliations:** 1 Department of Psychiatry, University of Cambridge; 2 Cognition and Brain Sciences Unit, University of Cambridge; 3 Department of Psychiatry, University of Cambridge; 4 Department of Psychiatry, University of Cambridge, and Wellcome Trust MRC Institute of Metabolic Science, Cambridge Biomedical Campus, Cambridge, United Kingdom; 5 Department of Psychiatry, University of Cambridge, and Cambridgeshire and Peterborough NHS Foundation Trust, Cambridge, United Kingdom

**Keywords:** perceptual priors, cognitive priors, psychosis, glutamate, ARMS

## Abstract

Alterations in the balance between prior expectations and sensory evidence may account for faulty perceptions and inferences leading to psychosis. However, uncertainties remain about the nature of altered prior expectations and the degree to which they vary with the emergence of psychosis. We explored how expectations arising at two different levels—cognitive and perceptual—influenced processing of sensory information and whether relative influences of higher- and lower-level priors differed across people with prodromal symptoms and those with psychotic illness. In two complementary auditory perception experiments, 91 participants (30 with first-episode psychosis, 29 at clinical risk for psychosis, and 32 controls) were required to decipher a phoneme within ambiguous auditory input. Expectations were generated in two ways: an accompanying visual input of lip movements observed during auditory presentation or through written presentation of a phoneme provided prior to auditory presentation. We determined how these different types of information shaped auditory perceptual experience, how this was altered across the prodromal and established phases of psychosis, and how this relates to cingulate glutamate levels assessed by magnetic resonance spectroscopy. The psychosis group relied more on high-level cognitive priors compared to both healthy controls and those at clinical risk for psychosis and relied more on low-level perceptual priors than the clinical risk group. The risk group was marginally less reliant on low-level perceptual priors than controls. The results are consistent with previous theory that influences of prior expectations in perceptions in psychosis differ according to level of prior and illness phase.

## General Scientific Summary

What we perceive and believe in any given moment will allow us to form expectations about what we will experience in the next. In psychosis, it is believed that the influence of these so-called perceptual and cognitive “prior” expectations on perception are altered, thereby giving rise to the symptoms seen in psychosis. However, research thus far has found mixed evidence, some suggesting an increase in the influence of priors and some finding a decrease. Here we test the hypothesis that perceptual and cognitive priors are differentially affected in individuals at risk for psychosis and individuals with a first episode of psychosis, thereby partially explaining the mixed findings in the literature. We indeed found evidence in favor of this hypothesis, finding weaker perceptual priors in individuals at risk but stronger cognitive priors in individuals with first-episode psychosis.

It has been hypothesized that the brain forms a model of the world by actively trying to predict it. These predictions are then updated iteratively by function of the prediction error. This hierarchical computational framework is usually referred to as predictive coding (Bar, 2007; Bastos et al., 2012; Clark, 2013, 2015; Friston, 2005, 2010; Hohwy, 2013; Knill & Pouget, 2004; Rao & Ballard, 1999). In this framework, the formation of delusional beliefs and hallucinatory experiences are proposed to be due to alterations in the cognitive and biological mechanisms of predictive coding (Adams, Stephan, Brown, Frith, & Friston, 2013; Fletcher & Frith, 2009).

While initial clinical studies documenting alterations in the way the expectation influences perception in psychosis are promising in demonstrating case-control alterations in various behavioral measures of predictive coding (e.g., Powers, Mathys, & Corlett, 2017; Shergill, Samson, Bays, Frith, & Wolpert, 2005; Teufel, Kingdon, Ingram, Wolpert, & Fletcher, 2010), it is already clear that there will be no straightforward unifying explanation of psychosis in simple terms of priors being “too strong” or “too weak” in general. Predictive processing theory envisions a highly interlinked (cortical) cognitive hierarchy, where different layers aim to predict the incoming input from lower layers (Bar, 2007; Bastos et al., 2012; Clark, 2013, 2015; Friston, 2005, 2010; Hohwy, 2013; Knill & Pouget, 2004; Rao & Ballard, 1999). Moving up the hierarchy, the predictions become more abstract, ranging from lower-level sensory prediction to higher-order beliefs about the environment. Therefore, it does not suffice to ask the question whether prior expectations are stronger or weaker in psychosis. Instead, in order to form a complete picture of the underlying mechanisms of psychosis, we need to look at the contribution of different types of prior expectations, including both sensory expectations and higher-level beliefs about the environment.

Recent influential predictive coding accounts of psychosis have emphasized that priors at low and high hierarchical levels may be differentially affected in psychotic illness. For example, Sterzer et al. (2018) concluded that “in contrast to weak low-level priors, the effects of more abstract high-level priors may be abnormally strong” in psychosis (p. 638). This postulate is mainly drawn through a combination of theoretical arguments and synthesis across diverse studies. To our knowledge, no study has yet demonstrated a combination of weak low-level perceptual priors and strong high-level cognitive priors in patients with psychosis, although Schmack and colleagues (2013) provided supportive evidence in a study of individual differences in healthy individuals. Those authors delineated priors at different hierarchical levels by manipulating what they referred to as perceptual priors and cognitive priors in two related experiments; they found that delusional ideation in health (sometimes termed delusion proneness) was associated with a decrease in the contribution of perceptual priors and an increase in the contribution of cognitive priors, highlighting the importance to separate the two (Schmack et al., 2013). Clearly, clinical studies testing the hypothesis of simultaneous weak low-level and strong high-level priors in psychotic illness are required, yet few have been attempted. One exception was another study from Schmack and colleagues, who found evidence against differential strengths of sensory and cognitive priors in schizophrenia (Schmack, Rothkirch, Priller, & Sterzer, 2017).

A further complexity is that cognitive and biological mechanisms of psychosis may be markedly different at different illness stages, adding nuance to the attractive, yet arguably overly simplistic, continuum model of psychosis. Previous reviews acknowledge that there may be evolving patterns of cognitive and/or physiological disturbances over time as psychotic illness develops (Adams et al., 2013; Fletcher & Frith, 2009; Heinz et al., 2019). In many cases, psychotic illness is heralded by the development of delusions (often delusional interpretations of hallucinations) after a prodromal period of hallucinatory experiences without delusional interpretation and/or delusional mood. In the context of weak low-level (sensory) priors and high precision of sensory prediction errors, delusions may emerge as a result of compensatory increases in the precision of high-level beliefs (i.e., enhanced high-level, cognitive priors; Adams et al., 2013; Heinz et al., 2019; Sterzer et al., 2018). It follows, then, that in the very early phases of psychosis, prior to the development of delusions, such compensatory increases in the precision of high-level beliefs may be yet to emerge. Although one previous study found alterations in the utilization of priors in individuals at clinical risk for psychosis (putatively in the prodrome) compared to controls (Teufel et al., 2015), this study did not include any patients with established psychotic illness; thus, none of the sample had developed delusions at the time of the experiment. Therefore, it remains unclear whether, or how, alterations in the use of higher- or lower-level priors change as psychotic illness emerges.

We acknowledge the vital importance of the range of previous studies exploring the contribution of prior expectation in perception in psychosis. However, here we argue that two important aspects of the predictive coding account have been largely neglected in empirical clinical studies: the contribution of different disease stages to the effect of prior expectations and the type of prior expectation. It is the aim of the present study to bring these two together by studying how different prior expectations are affected throughout individuals at risk for psychosis and individuals who recently had an episode of psychosis.

In order to test the hypothesis that sensory and cognitive priors are differently used depending on the stage of psychosis, we designed two novel auditory perception paradigms: one testing the influence of lip movements on auditory perception (perceptual priors) and a second testing the influence of learned written word-sound associations on auditory perception (cognitive priors). We gathered data on these two paradigms in two patient groups—individuals at elevated clinical risk for psychosis and individuals who recently had their first episode of psychosis—and compared them to a group of healthy controls. Help-seeking individuals who are at risk for psychosis usually have subclinical psychotic symptoms that are not severe or frequent enough to warrant a clinical diagnosis but are at considerably raised risk of developing a psychotic illness in the short to medium term (Yung, Yuen, Phillips, Francey, & McGorry, 2003). Studying these early stages of illness may help us to understand the mechanisms underlying the emergence of a psychosis by examining which aberrancies precede psychosis and might, therefore, be predictive of developing psychosis.

The first paradigm (from now on *perceptual priors task*) assesses the influence of lip movements on auditory perception. Lip movements have been shown to influence auditory perception. McGurk and MacDonald (1976) showed that when individuals were presented with an auditory (“Ba”) phoneme in combination with lip movements pronouncing (“Ga”), most individuals perceive a mixture between the two (“Da”). This effect has become known as the McGurk illusion (McGurk & MacDonald, 1976). Studies of the neural mechanisms underlying the influence of lip movements on auditory perception provide support for the Bayesian framework in that lip movements are suggested to constitute a prior expectation with respect to the incoming auditory signal (Arnal, Wyart, & Giraud, 2011; Blank & Davis, 2016). One previous study of mainly male, middle-aged adults with chronic schizophrenia documented a diminishment in perceiving the McGurk illusion, relying more on the auditory input; this finding was associated with illness chronicity (White et al., 2014). Pearl et al. (2009) also studied the McGurk illusion in schizophrenia, finding mixed results: adolescents with schizophrenia, but not adults with schizophrenia, showed a diminished illusory effect. Schizophrenia has been associated with a diminished ability in using lip movements in aiding auditory discrimination, suggesting aberrancy in the ability to integrate the two sources of information (de Gelder, Vroomen, Annen, Masthof, & Hodiamont, 2003; Myslobodsky, Goldberg, Johnson, Hicks, & Weinberger, 1992; Ross et al., 2007; Szycik et al., 2013). However, it remains unclear whether the influence of prior information in auditory perception is altered in the early stages of psychosis as no previous first-episode psychosis study or study of people with prodromal symptoms of psychosis has been conducted. The purpose of the perceptual priors task was to measure precisely how much lip movements influence what participants hear by using a staircase procedure (Cornsweet, 1962), in which the balance between two sounds was changed in predefined steps, providing more fine-grained measures of individual susceptibility to the illusion than in previous clinical studies.

The second paradigm (from now on *cognitive priors task*), assesses the influence of learned written word-sound associations on auditory perception. The impact of learned associations on auditory perception has been shown in sensory conditioning, where one stimulus functions as a predictor for an auditory stimulus that is otherwise difficult to detect. In these early experiments, participants were asked to identify auditory stimuli based on a visual cue. Sometimes the participants reported perceiving an auditory stimulus when only presented with the visual cue as the brain predicted an auditory stimulus on the basis of the cue (Agathon & Roussel, 1973; Brogden, 1947; Ellson, 1941; Kot & Serper, 2002; Powers et al., 2017; Warburton, Wesnes, Edwards, & Larrad, 1985). Previous research found that this omission effect is stronger in individuals with hallucinations (Kot & Serper, 2002; Powers et al., 2017), suggesting an increase in the influence of learned *cognitive* expectation on auditory perception in psychosis, in contrast to the diminishment in the influence of *sensory* expectations in schizophrenia discussed earlier. However, to date, no study has explored the influence of learned cognitive expectations in individuals at risk for psychosis and compared it to the influence of sensory expectations on perception.

We recognize that the sensory and cognitive priors tasks are, strictly speaking, not able to estimate the relative precision and mean of the prior expectations and sensory evidence for each participant directly. Instead, we assume, based on Bayesian theories of the brain, that perception is a function of the precision and mean of the prior and sensory evidence. Therefore, rather than estimating the precision and mean for the prior and sensory evidence separately, we infer the relative contribution of prior information and sensory evidence; for the remainder of this paper, we term this the *relative strength* of the sensory and cognitive priors. Reconciling the exact level of priors used in the current experiment in relation to the exact level of priors used in previous experiments in schizophrenia spectrum patients is not trivial. However, this is not central to our experiment. Our aim is to examine the effects of two different levels of priors on a given process at different stages of psychosis.

Another issue currently understudied relates to the neurobiological underpinnings of alterations in the contribution of prior expectations in perception. Changes in glutamate levels have been associated with schizophrenia and individuals at risk for psychosis (Egerton et al., 2014; Marsman et al., 2013; Merritt, Egerton, Kempton, Taylor, & McGuire, 2016; Treen et al., 2016), including in the cingulate cortex, where there is evidence of excessive glutamate in early illness stages, possibly progressing to reductions in later stages (Kumar et al., 2018; Merritt et al., 2016). It remains unclear to what extent glutamate levels in the brain relate to predictive coding mechanisms putatively mediating psychosis, in spite of various theoretical arguments and extrapolations from preclinical experiments (Corlett, Honey, Krystal, & Fletcher, 2011; Sterzer et al., 2018). Notably, the anterior cingulate cortex (ACC) has been associated with processing uncertainty (Rushworth & Behrens, 2008) and precision weighting of information in health and psychosis (Cassidy et al., 2018; Haarsma et al., 2019; Katthagen et al., 2018). Thus, alterations in glutamate levels in the ACC might alter the precision of prior information, thereby changing the degree to which priors influence perception. Therefore, we explored this issue by measuring magnetic resonance spectroscopy (MRS) glutamate levels in the anterior cingulate cortex and relating these measurements to the contribution of prior expectations in the different experimental groups. Our study was not powered to provide definitive results relating glutamate measures to our predictive coding measures, the latter being of primary interest here. Nevertheless, we report preliminary, exploratory analyses that may be hypothesis generating and could provide the basis for power calculations for future studies combining MRS with behavioral data in patients.

In summary, we used a cross-sectional design to study altered use of prior expectations in auditory perception in individuals at risk for psychosis, first-episode psychosis, and controls. We expected to find differences in the balance between the use of prior expectations and sensory input depending on the origin of the prior expectation (sensory vs. cognitive) and disease stage (at risk vs. first-episode psychosis). Specifically, we expected that at early stages of psychosis (clinical risk), patients would make relatively stronger use of sensory input than prior expectations relative to controls and individuals with a full manifestation of illness (first-episode psychosis) but that in those with first-episode psychosis, patients would rely more on cognitive priors relative to sensory input compared to controls and individuals at risk for psychosis. A secondary hypothesis was that cortical glutamate levels would be related to changes in the usage of sensory and cognitive priors.

## Method

### Participants

Participants with first-episode psychosis (FEP; *n* = 30, average 24.8 years, six female) or at-risk mental state patients (ARMS; *n* = 29, average 21.5 years, eight female) were recruited from the Cambridge Early Intervention Service North and South. In addition, ARMS patients were recruited from a help-seeking, low-mood, high-schizotypy subgroup following a latent class analysis on the Neuroscience in Psychiatry Network cohort (Davies, 2017) or through advertisement via posters displayed at the Cambridge University counseling services. Individuals with FEP or at-risk mental states for psychosis met FEP or ARMS criteria on the Comprehensive Assessment for the At-Risk Mental State (CAARMS) Interview. All FEP participants had current delusions or previous delusions in the case of those with partial or recent recovery. Healthy volunteers (healthy control sample or HCS; *n* = 32, average 22.6 years, 15 female) without a history of psychiatric illness or brain injury were recruited as control subjects. Healthy volunteers did not report any personal or family history of neurological, psychiatric, or medical disorders. All participants had normal hearing and normal or corrected to normal vision. All participants gave informed consent. The study was approved by the West of Scotland (REC 3) Ethical Committee. See Table 1 for details on demographics and symptom scores. Three ARMS patients and 17 FEP patients were receiving antipsychotic medication.[Table-anchor tbl1]

### Questionnaires and Interviews

We used the Cardiff Abnormal Perceptions Scale (CAPS, Bell, Halligan, & Ellis, 2006), Peters Delusion Index Scale (PDI, Peters, Joseph, & Garety, 1999), CAARMS (Yung et al., 2003), and Positive and Negative Symptoms Scale (PANSS, Kay, Opler, & Lindenmayer, 1989) to assess “caseness,” symptom severity and frequency. Both the total scores for the CAPS and PDI and the subscales of the CAPS and PDI are reported in Table 1. For the PDI and CAPS, the participants were required to give a “yes” or “no” answer to a particular question. In case of a “yes” answer, three subscales were filled in, which utilized a 5-point Likert scale. The CAARMS and PANSS are semistructured interviews, where the interviewer rates severity of various types of psychotic and other psychiatric symptoms.

### Magnetic Resonance Spectroscopy

A subset of participants was scanned on a Siemens Prisma 3T scanner at the cognition brain sciences unit in Cambridge. The spectroscopy scan was part of a larger MRI protocol that contained, in addition, two fMRI protocols and a structural scan totaling 90 min. The structural scan was used to plan the MRS voxel. A 15-mm isotropic voxel was placed carefully in the anterior cingulate cortex. A point resolved spectroscopy sequence was used to assess glutamate levels, with a repetition time of 1,880 ms and echo time of 30 ms. One hundred fifty water-suppressed acquisitions were collected in addition to 16 unsuppressed acquisitions. Data was analyzed in LCModel. MRS data was successfully collected from 18 healthy controls, 19 ARMS, and 14 FEP patients.

### Experiment 1: Providing Perceptual Priors

**Figure 1 fig1:**
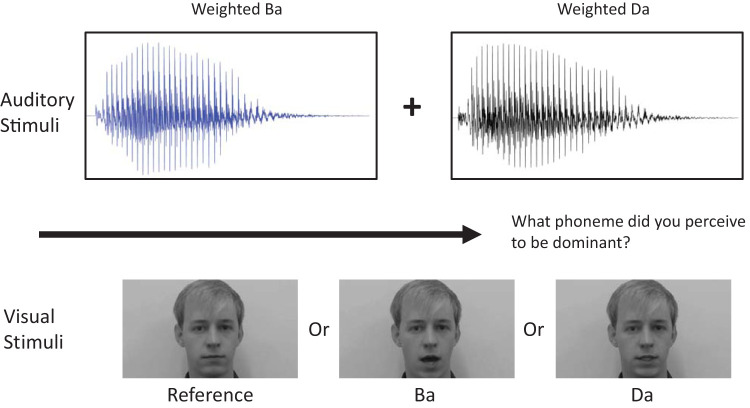
Procedure of the sensory prior task. The participant was presented a mixture of the phonemes “Ba” and “Da” (above), which co-occurred with either a still face (reference condition) or lip movements pronouncing “Ba” or “Da.”

In the present study, auditory stimuli were presented that contained varying proportions of the phoneme “Ba” or “Da” (see Figure 1). The balance between the two stimuli always adds up to one. The contribution of the stimulus “Ba” is denoted as ω^Ba^, which stands for “the weight of ‘Ba’”. The proportion of ω^Da^ can be derived from ω^Ba^ as 1 − ω^Ba^ = ω^Da^. Henceforth, the notation ω^Ba^ will be used to indicate what exactly was presented to participants in terms of auditory stimulus.[Fig-anchor fig1]

#### Training phase

The task started with a training phase, the purpose of which was to familiarize the participants with the auditory stimuli. Here they were presented with a still face in combination with an auditory stimulus consisting of a stimulus ω^Ba^ = .8 or ω^Ba^ = .2. They were then asked to report which sound they believed was dominant, after which they received feedback (correct/incorrect). The training was completed as soon as participants reported the correct answer four times for each stimulus. All participants identified the phonemes correctly.

#### Testing phase

During the testing phase, the participants were presented with an auditory stimulus consisting of a mix between the sounds “Ba” and “Da” (as described earlier), which simultaneously occurred with a visual stimulus consisting of a black and white male face. The face would pronounce either “Ba” or “Da” (lip movement condition) or the face would remain still (the reference condition). All three conditions were presented in a pseudorandomized order such that all three conditions were presented in a random order before one of the conditions was presented again. The participants were instructed to keep looking at the lips of the face throughout the task but were asked to report what phoneme was dominant in the auditory stimulus by pressing one of four buttons indicating the level of certainty and the perceived phoneme.

**Figure 2 fig2:**
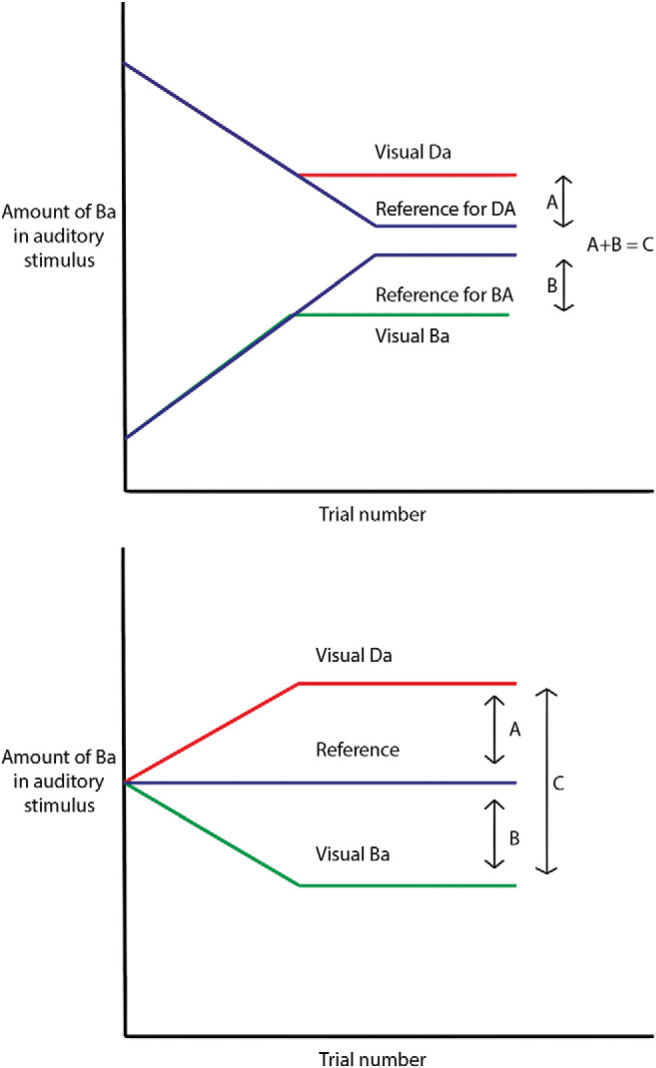
Schematic representation of a staircase in the perceptual priors task (upper panel) and cognitive priors task (lower). The experiment adjusted the balance between “Ba” and “Da” during the experiment in favor of the nonreported stimulus (slope line), ensuring convergence to a subject threshold (flat line). The distance A indicates the strength of the “Da” prior, whereas B indicates the strength the “Ba” prior. C is a total measure of prior strength irrespective of the specific prior presented.

**Figure 3 fig3:**
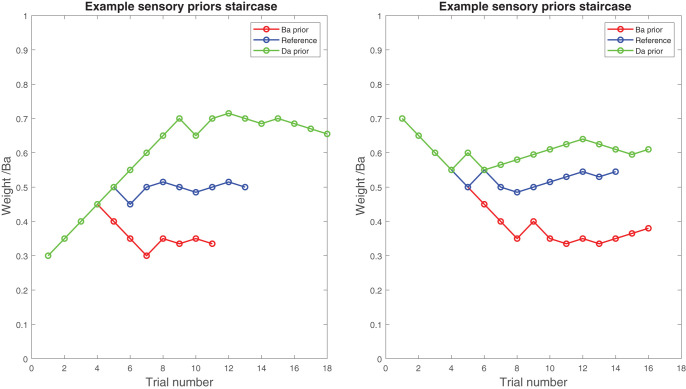
Example of the staircase procedure. All six of the conditions are represented here. The left figure shows the three visual conditions (green/light gray: visual Ba condition, blue/dark gray: Reference condition, red/middle gray: visual Da condition) where the staircase started at ω^Ba^ = .3, whereas the right figure shows the three visual conditions where the staircase started at ω^Ba^ = .7.

During the main task, the balance between the “Ba” and “Da” phonemes was changed in a stepwise fashion. That is, when the participant reported the sound “Ba” to be dominant in, for example, the reference condition, then the next time that condition came up, the balance between the sounds “Ba” and “Da” would be shifted in favor of the nonreported phoneme—in this case, “Da.” By following this procedure, the task would converge toward a point where the participant would find it difficult to distinguish which of the phonemes was dominant in the auditory stimulus. This point is referred to as the perceptual indifference point. In the reference condition, where no lip movements were presented, we expected the perceptual indifference point to converge on a stimulus that contains .5 of “Ba” and .5 of “Da.” However, when lip movements, for example pronouncing “Ba,” were presented to bias perception toward the prior expectation, we expected that the task would converge upon an indifference point that contained less auditory “Ba” and more auditory “Da.” In other words, more auditory “Da” was needed to overcome the influence that the “Ba” lip movements had (see Figure 2, top panel, for a schematic representation of the perceptual staircase experiment and Figure 3 for an example of a staircase).[Fig-anchor fig2][Fig-anchor fig3]

For each of the three conditions (reference, “Da,” and “Ba”), the perceptual indifference point was assessed twice: once where the auditory stimulus started with a dominant “Ba” stimulus (ω^Ba^ = .7, ω^Da^ = .3) and once where “Da” was dominant (ω^Ba^ = .3, ω^Da^ = .7). This created six conditions, which were presented to the participant in pseudorandom order. A condition was completed when either one of two criteria was met. First, in the majority of cases, a perceptual indifference point was reached, which was defined as having made six switches in perceiving one stimulus over the other (e.g., previously perceiving “Ba” on trial T-1 and perceiving “Da” on trial T-0, indicating the balance between the two auditory stimuli is close to the participants perceptual indifference point). Second, a condition was completed when the participant indicated that the sound “Ba” or “Da” was 100% dominant in the auditory stimulus (e.g., a participant perceived “Da,” even though the stimulus was 100% “Ba,” which could happen when the visual priors are dominating perception). In the second case, this would technically not be an indifference point. However, for the remainder of this article, we will refer to it as such for the sake of simplicity. The priors dominated perception only in a small minority of cases (see Results). A condition was aborted when 30 trials had been presented to avoid the task from taking too long. This did not change the way the effect of the prior was calculated. In order to test for group and condition differences in the amount of trials needed to reach an indifference point and a possible interaction, we used a mixed ANOVA with group as the between-subjects factor and visual condition as the within-subject factor.

At the beginning of the staircase, the balance between “Ba” and “Da” was changed in steps of .05. After the first switch, the balance was changed in steps of .015. This procedure ensured that the first switch was reached quickly. Thereafter, the staircase became more sensitive so that the perceptual indifference point could be assessed more precisely. The strength of each of the visual priors was calculated separately as the difference between the perceptual indifference point of the visual prior condition and the reference condition (see Figure 2, upper panel: A and B). The total strength of the visual priors was calculated as the distance between the indifference points of both sensory prior conditions (see Figure 2, upper panel: C).

### Experiment 2: Providing Cognitive Priors

#### Training phase

The cognitive priors task was designed to measure how much a learned cue would influence what participants hear. During the training phase, participants learned the association between the letters “BA” and the phoneme “Ba,” and vice versa for “DA.” In 75% of the training trials, the letters “BA” or “DA” were presented 500 ms prior to hearing the auditory stimulus, which consisted of ω^Ba^ = .3 and ω^Da^ = .7 when preceded by the letters “DA” or ω^Ba^ = .7 and ω^Da^ = .3 when preceded by the letters “BA,” making the letters predictive of the auditory stimuli. In the other 25% of the trials, no sound was presented following the letters. Here the participants were asked to report what they expected to hear. The training was complete as soon as the participants indicated eight times that they expected to hear “Ba” following the letters “BA” and “Da” following the letters “DA.”

#### Testing phase

**Figure 4 fig4:**
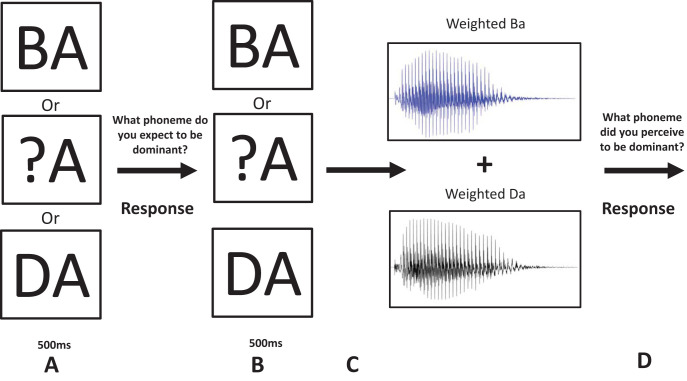
Procedure of the experimental phase of the cognitive prior task. A: One of the three sets of letters was presented to the participant to indicate what sound was most likely to occur according to the training phase. B: Participants were required to indicate which phoneme they believed to be most likely presented. C: The participant was again presented with one of the three letters (the same as in A) and was presented with the mixed phoneme 500 ms later. D: After the presentation of the sound, the stimuli were removed from the screen and the participant was required to indicate what phoneme they perceived to be dominant.

The cognitive priors task is similar to the perceptual priors task in that participants were instructed to report which sound they believed to be dominant under different prior expectations. However, this time the prior expectations came from learned written word-sound associations. Again, the main task consisted of three conditions, a cognitive prior “BA” and “DA” condition and a reference condition, which consisted of “?A.” Each trial started with the presentation of the letters “BA,” “DA,” or “?A.” After seeing “BA” or “DA,” participants were asked which phoneme they expected to perceive, which they indicated using one of four buttons indicating the perceived phoneme and certainty like in the perceptual priors task. The participants were only asked to indicate their prediction following seeing the letters “BA” and “DA” but not after seeing “?A.” By making a conscious prediction regarding the upcoming stimulus, the use of the cognitive prior could be validated. In the reference condition, no reliable prediction could be generated as both options were equally likely. The auditory stimulus was presented 500 ms after they made a decision or the reference stimulus had been presented. Subsequently, participants indicated what they perceived to be the dominant stimulus (see Figure 4).[Fig-anchor fig4]

Again, the balance between the auditory phonemes “Ba” and “Da” was shifted in favor of the nonreported stimulus in a stepwise fashion. However, in contrast to the perceptual priors task, each condition was presented once for each cognitive prior “BA” and “DA” instead of twice. Within the cognitive “BA” prior condition, the staircase started at ω^Ba^ = .7 and ω^Da^ = .3, meaning the auditory stimulus was relatively clearly a “Ba” sound. The same was true for the cognitive “DA” prior condition, where the staircase started at ω^Ba^ = .3 and ω^Da^ = .7, meaning the auditory stimulus was relatively clearly a “Da” sound. This matching of the auditory stimulus to the cognitive prior condition at the beginning of the staircase was done to reaffirm the association between the prior and the sound; otherwise, the association between the cue and sound could have been lost immediately in the beginning of the staircase. Note that if we were to compare the difference in perceptual indifference points in the two cognitive prior conditions, we would have a confound as the staircases for the two cognitive prior conditions started at different intensities, explaining any differences between the two conditions. Therefore, we introduced two reference conditions to which the prior conditions can be compared, getting rid of the confound. These consisted of the letters “?A.” One of the conditions had a staircase starting at ω^Ba^ = .7 and ω^Da^ = .3 so it could be directly compared to the cognitive “BA” prior; the other started at ω^Ba^ = .3 and ω^Da^ = .7 so it could be directly compared to the cognitive “DA” prior. As in the first task, at the beginning of the staircase procedure, the balance between “Ba” and “Da” was changed in steps of .05. Then, after the first switch, the balance was changed in steps of .015.

In total, the cognitive priors task consisted of four conditions: a “BA” and a “DA” condition, a reference condition for “BA,” and a reference condition for “DA.” The order of the condition per participants was pseudorandomized. In each condition, a perceptual indifference point was assessed.

The perceptual indifference point for each condition was quantified by taking the average of ω^Ba^ at the last two switches. We also briefly report the results for taking the final four switches to demonstrate this does not influence the results substantially. In order to quantify the strength of each prior, these perceptual indifference points were subtracted from their reference condition, and the total cognitive prior strength was calculated by adding the strength of separate priors (see Figure 2, lower panel).

### Stimuli, Apparatus, and Procedure

Participants completed two tasks: the perceptual priors task first and the cognitive priors task second. Each task was performed on a 13-inch retina MacBook Pro, and each lasted about 10 min on average. Participants wore Sennheisser headphones to ensure optimal hearing. Both the “Ba” and the “Da” stimuli had an intensity of 68 dB. All participants reported perceiving the auditory stimuli clearly. The experiment was conducted in an environment with minimal background noise, ensuring minimal distraction of the participant (<15 dB).

Psychtoolbox-3 was used to design the experiment. The auditory stimulus in both the perceptual priors task and the cognitive priors task consisted of a mixture of a natural speech male voice “Ba” phoneme and a “Da” phoneme. The auditory stimulus was created by multiplying the auditory spectrum of the “Ba” stimulus by a weighting factor ω^Ba^. This was then added to a weighted auditory spectrum of “Da” (where ω^Da^ = 1 − ω^Ba^), ensuring the total of auditory stimulus to always be 1 [stimulus = (ω^Ba^ × Ba) + (ω^Da^ × Da)].

### Analyses

Since this is a novel paradigm, we first wanted to establish whether the variables of interest were reliable in the sense that two separate measurements of the variable were highly correlated. Since we assessed the perceptual indifference points twice in each condition, we were able to test the correlation between two separately obtained measurements, giving an indication of their reliability. We tested the reliability of two separate variables. First, we tested the reliability of the indifference points in the condition without a perceptual prior, which should give an indication of the reliability of the staircase method. Second, we tested the reliability of the strength of the perceptual priors, which should give an indication of the reliability of the method to measure the influence of lip movements on auditory perception. Furthermore, we tested whether the perceptual and cognitive priors were correlated with each other. Due to nonnormality of the cognitive priors task, a Spearman correlation was used to assess this.

One-tailed paired *t* tests were used to test for a main effect of whether the lip movements shifted the perceptual indifference points in the expected direction compared to the reference condition. This was done for both the sensory and cognitive priors tasks. In order to test the hypothesis that perceptual priors and cognitive priors were different across groups, we computed the influence of the prior for each individual as described earlier and used a one-way ANOVA with two-tailed post hoc Bonferroni-corrected *t* tests if applicable. Furthermore, a Kruskal Wallis nonparametric ANOVA was used with cognitive prior data, with Bonferroni-corrected nonparametric post hoc *t* tests. We also report the results of Bayesian statistical tests in relation to the group differences using JASP. We report effect sizes for the key statistical tests—that is, effect of group on prior strength. We report Cohen’s *d* for *t* tests and η^*2*^ for the one-way ANOVAs. All effect sizes were calculated based on parametric tests.

## Results

### Perceptual Priors Task

#### No difference between groups in the amount of trials needed to assess perceptual indifference point

On average, participants required 18.9 trials to reach a perceptual indifference point across all conditions. We found no overall effect of group on the trials needed to reach a perceptual indifference point, *F*(2, 87) = .262, *p* = .77; HCS = 19.1, *SE* = 0.5; ARMS = 19.1, *SE* = 0.6; FEP = 18.6, *SE* = 0.4. However, we did find an effect of prior condition, *F*(2, 174) = 17.1, *p* < .001, needing fewer trials in the visual reference condition (17.3, *SE* = 0.3) than in the visual “BA” (18.9, *SE* = 0.4) and visual “DA” condition (20.7, *SE* = 0.54). Importantly, we found no group by condition interaction, *F*(4, 174) = .456, *p* = .77. Thus, the patient groups did not differ in terms of the trials needed to reach indifference points.

#### Individual perceptual indifference points can be estimated reliably

**Figure 5 fig5:**
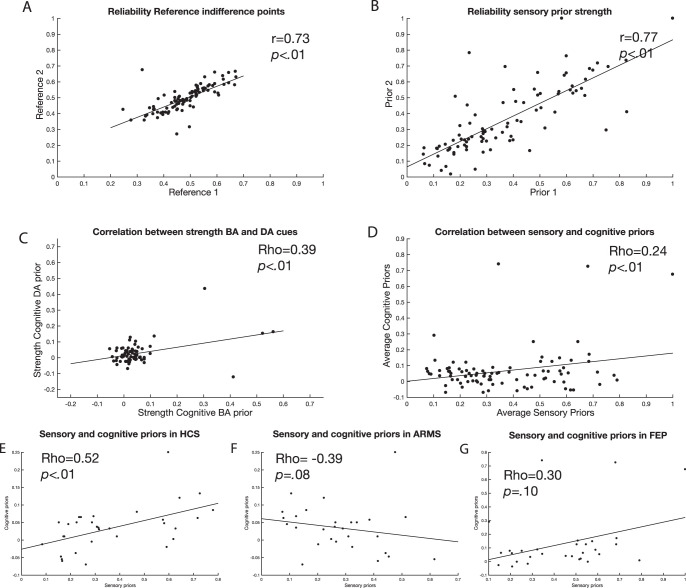
Correlations testing the reliability of the experiment are presented here. A: Reliability of the perceptual indifference point in the reference condition. B: Reliability of the strength of perceptual priors. C: Correlation between the effect of cognitive “Ba” stimulus and the cognitive “Da” stimulus. D: Correlation between sensory and cognitive priors. E–G: Relationship between cognitive and sensory priors for each experimental group. Whereas healthy controls and FEP show a positive correlation, ARMS show a negative correlation. We calculate Spearman correlations but include linear fit lines for display purposes. HCS = healthy control sample; ARMS = at-risk mental state patients; FEP = first-episode psychosis.

The perceptual indifference point for each visual condition was assessed twice in the perceptual priors task. As this is a novel task, we tested whether these simultaneously assessed indifference points correlated strongly as that would give us an indication of the reliability of the measurement. First, we correlated the indifference points in the condition where no priors were presented (the reference condition). Across groups, the correlation was *r* = .73. Separately, it was *r* = .83 for HCS, *r* = .76 for ARMS, and *r* = .55 for FEP (all *p*s < .01). The correlation between the two reference points was significantly higher in the HCS group compared to the FEP group (Fisher’s *r*-to-*z* transformation: *p* = .033) but not between other groups (all *p*s > .2). Second, in a similar fashion, we assessed how strongly the effect of the perceptual priors was correlated across the two simultaneously assessed staircases. The reliability of the strength of the perceptual priors across groups was *r* = .78. Separately, it was *r* = .88 for HCS, *r* = .79 for ARMS, and .69 for FEP (all *p*s < .01; see Figure 5). The differences in correlations between perceptual priors were not significantly different, *p* > .2. For the remainder of the analyses, we averaged the perceptual indifference points for each visual condition (“Ba,” “Da,” and reference) and the estimation of the sensory prior strength (see Figure 2).[Fig-anchor fig5]

#### Perceptual priors shifted the perceptual indifference points in the expected direction

**Figure 6 fig6:**
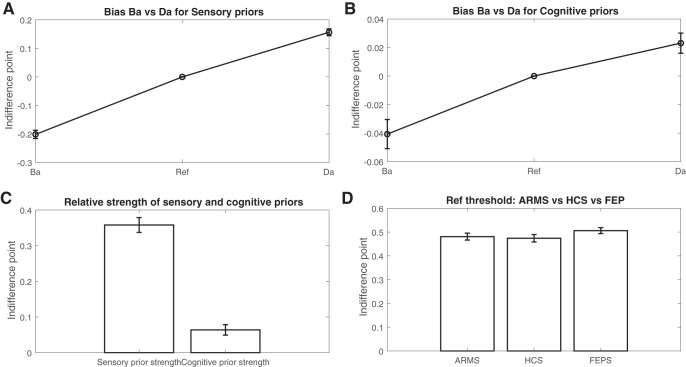
Main effects of the sensory and cognitive priors are presented here. A: Relative shift in perceptual indifference points under different sensory prior conditions (lip movements pronouncing “Ba” or “Da”) compared to reference condition (still lips). B: Relative shift in perceptual indifference points under different cognitive prior conditions (the letters “BA” and “DA”) compared to reference condition (“?A”). C: Relative strength of perceptual priors and cognitive priors. D: The perceptual indifference points in the reference conditions per group (effect of no interest). Error bars represent standard error of the mean. HCS = healthy control sample; ARMS = at-risk mental state patients; FEP = first-episode psychosis.

We tested whether the perceptual priors shifted the perceptual indifference points in the expected directions compared to the reference condition. On average, across all groups taken together, “Ba” lip movements lowered the value of ω^Ba^ in the perceptual indifference point by .21, 95% CI [.18, .23], *t*(89) = 14.0, *p* < .0001. In contrast, “Da” lip movements increased the value of ω^Ba^ in the perceptual indifference point by .16, 95% CI [.14, .18], *t*(89) = 13.2, *p* < .0001, on average. When comparing the relative strength of the “Ba” and “Da” lip movements, we found a significant difference, *t*(178) = 2.29, *p* = .022, indicating a slightly stronger effect of “Ba” lip movements than “Da” (Figure 6A).[Fig-anchor fig6]

#### The perceptual indifference point in the reference condition was equal across groups

Analyzing group differences, the perceptual indifference point in the reference condition was a variable of no interest as it merely reflects a personal preference for either the auditory “Ba” or “Da” stimulus. Indeed, the average perceptual indifference point in the reference condition across groups in reference groups was equal, M^HCS^ = .48, *SE*^HCS^ = .02; M^ARMS^ = .49, *SE*^ARM^ = .01; M^FEP^ = .51, *SE*^FEP^ = .01; *F*(2, 88) = 1.02, *p* = .36 (Figure 6D). This shows that there were no differences in preference for either the auditory “Ba” or “Da” stimulus between the different groups.

#### Perceptual priors were significantly lower in ARMS compared to FEP

**Figure 7 fig7:**
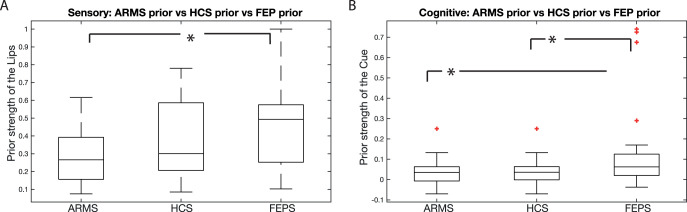
The effects per group are presented here. A: The effect of perceptual priors across groups. B: The effect of cognitive priors across groups. HCS = healthy control sample; ARMS = at-risk mental state patients; FEP = first-episode psychosis. * *p* < .05.

To test whether the perceptual priors were significantly different across groups, we conducted a one-way ANOVA. We indeed found evidence for a difference across groups, *F*(2, 88) = 5.32, *p* = .007, effect size η^*2*^ = .11 (Figure 7A, 7C). Bonferroni-corrected post hoc *t* tests revealed a significant difference between ARMS (M^ARMS^ = .28, *SE*^ARMS^ = .03) and FEP (M^FEP^ = .44, *SE*^FEP^ = .04), *p* = .005, effect size *d* = .89, 95% CI [.46, 1.32], but not between healthy controls (M^HCS^ = .37, *SE*^HCS^ = .04) and ARMS, *p* = .20, effect size *d* = −.51, 95% CI [.01, 1.01], or FEP, *p* = .44, effect size *d* = .34, 95% CI [−.13, .85]. We tested whether changing the amount of switch points that were used to calculate the indifference point changed the results. When we changed this from two to four, we found the same (slightly stronger) effect, *F*(2, 88) = 5.72, *p* = .005; ARMS vs. FEP: *p* = .002; ARMS vs. HCS: *p* = .12; HCS vs. FEP: *p* = .24.[Fig-anchor fig7]

Furthermore, we analyzed the perceptual prior data in a Bayesian fashion. For this section, we use Jeffreys’s (1961) suggested evidence categories for the Bayes factor (BF). We found that an ANOVA revealed moderate evidence in support for a difference across groups (BF = 6.3). Independent sample *t* tests revealed anecdotal evidence in favor of a difference between ARMS and healthy controls (BF = 1.4) but anecdotal evidence in favor of no difference between healthy controls and FEP (BF = 1.8). There was strong evidence for a difference between ARMS and FEP (BF = 26.1; Figure 7A, 7C).

In some participants the perceptual priors were so strong that they dominated auditory perception completely. The “Ba” perceptual prior dominated perception completely in 4/32 HCS, 0/29 ARMS, and 5/31 FEP participants, whereas the “Da” perceptual prior dominated perception completely in 5/32 HCS, 2/29 ARMS, and 11/31 FEP participants. In one FEP participant, both the “Da” and “Ba” lip movements completely dominated perception.

### Cognitive Priors Task

#### FEP needed on average an extra trial to finish the training phase

We first tested whether the different experimental groups differed in the amount of trials needed to end the training using an ANOVA. The groups differed significantly in the number of trials needed, *F*(2, 88) = 3.34, *p* = .040. The HCS group and the ARMS group required on average 8.7 trials and 8.8 trials, respectively, before the training was finished, whereas the FEP required on average 9.9 trials.

#### No difference between groups in the amount of trials needed to assess perceptual indifference point

During the actual experiment, the participants generally required 18.5 trials to reach a perceptual indifference point across all conditions. We found no overall effect of group on the trials needed to reach a perceptual indifference point, *F*(2, 88) = .44, *p* = .64; HCS = 18.5, *SE* = 0.6; ARMS = 18.9, *SE* = 0.6; FEP = 18.1, *SE* = 0.6. However, we did find an effect of prior condition, *F*(2, 88) = 3.56, *p* = .033, needing significantly fewer trials in the “DA” condition (17.6, *SE* = 0.5) than in the visual “BA” (19.5, *SE* = 0.5), *t*(180) = 2.63, *p* = .018, but not the reference condition (18.3, *SE* = 0.5), *t*(180) = 1.08, *p* = .56 (Bonferroni corrected). Importantly, we found no group by condition interaction, *F*(4, 176) = .27, *p* = .90. Thus, the patient groups did not differ in terms of the trials needed to reach indifference points.

#### Cognitive priors shifted the perceptual indifference points in the expected direction

In order to assess the main effect of cognitive priors, each perceptual indifference point of the two cognitive prior conditions was subtracted from its own reference condition. We found that the cognitive “BA” prior lowered the value of ω^Ba^ by .042, *z* = −5.2, *p* < .0001, and for the cognitive “DA” prior the value of ω^Ba^ was increased by .027, *z* = 3.7, *p* = .0002. This shows that there was indeed a main effect of cognitive priors on perceptual indifference points. The relationship between effect of “BA” and “DA” priors is shown in Figure 5C. For the remainder of the analyses, the degree of influence of the “BA” and “DA” cognitive priors were added together and averaged in order to create a single measure of cognitive prior strength (see Figure 6B).

#### Effect of cognitive priors in the FEP group was significantly higher than the ARMS and controls

We used a nonparametric ANOVA that is robust against Type I errors in nonnormally distributed data. The differences between the average strength of the cognitive priors was significant (independent samples Kruskal-Wallis test: *p* = .023, effect size η^*2*^ = .11). Using a post hoc Bonferroni-corrected Wilcoxon’s rank sum test, we found stronger usage of cognitive priors in the FEP group compared to both the HCS group, *z* = 2.35, rank sum = 840, *p* = .037, effect size *d* = .64, 95% CI [.11, 1.17], and the ARMS group, *z* = 2.35, rank sum = 714, *p* = .037, effect size *d* = .62, 95% CI [.10, 1.14], but there was no significant difference between the HCS group and the ARMS group *p* > .5. We tested whether changing the amount of switch points that were used to calculate the indifference point changed the results. When we changed this from two to four, we found the same (slightly stronger) effect, FEP vs. HCS: *p* = .015, FEP vs. ARMS: *p* = .016, HCS vs. ARMS: *p* > .5.

We also analyzed the cognitive prior data in a Bayesian fashion and found that an ANOVA revealed moderate evidence in support for a difference across groups (BF = 7.5). Independent sample *t* tests revealed moderate evidence in favor of no difference between ARMS and healthy controls (BF = 3.5) but moderate evidence in favor of a difference between healthy controls and FEP (BF = 3.5). There was also anecdotal evidence for a difference between ARMS and FEP (BF = 2.8; Figure 7B, 7D). Although we had no evidence that the extreme values represent measurement error, we analyzed the results having excluded outliers in all three experimental groups (one in HCS, one in ARMS, three in FEP). We found similar results (two-sample *t* test adjusted for multiple comparisons; averaging over the final two switch points: HCS vs. FEP: *p* = .035, ARMS vs. FEP: *p* = .038; final four switch points: HCS vs. FEP: *p* = .050, ARMS vs. FEP: *p* = .051).

There were only a few FEP participants where the cognitive priors completely dominated perception. There was one FEP participant for whom the “BA” prior completely dominated perception and two other FEP participants for whom the “DA” prior completely dominated perception, with no occurrences in ARMS or HCS. There were no participants for whom both the “BA” and “DA” cues completely dominated perception.

#### Perceptual priors had a stronger effect on perception than cognitive priors and were differently correlated across groups

Finally, we analyzed whether the strength of the priors was different between tasks. This was indeed the case, showing a stronger effect of perceptual priors (.37) compared to the cognitive priors across all groups (.07), *t*(90) = −14.34, *p* < .0001, effect size *d* = 1.5, 95% CI [1.8, 1.2] (Figures 5D, 6C). Subsequently, we tested whether the strength of cognitive and perceptual priors was correlated using a Spearman correlation. This was indeed the case (ρ = .24, *p* < .02). When exploring the correlations separate for each group, we found a negative (trend-level) correlation in the ARMS group (ρ = −.33, *p* = .08) and positive correlations in the HCS (ρ = .52, *p* = .002) and (trend level) in the FEP group (ρ = .30, *p* = .10). Using a Fisher’s *r*-to-*z* transformation, we found that the relationship was significantly different for the ARMS group compared to the healthy control group (*z* = −3.28, *p* = .001) and FEP group (*z* = −2.25, *p* = .024). The correlation between healthy controls and FEP was not significantly different (*z* = 1.0, *p* = .31). As these findings constituted secondary analyses, they were not properly controlled for multiple comparisons. When controlling for multiple tests, only the relationship in the healthy control group remained significant.

#### Glutamate levels correlated with cognitive priors in HCS and perceptual priors in FEP

**Figure 8 fig8:**
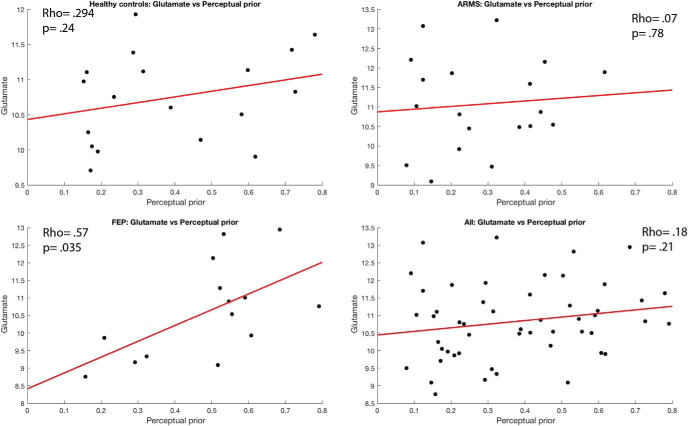
Correlations between perceptual prior strength and glutamate levels for all groups. We report Spearman’s correlations but plot linear fits for display purposes.

**Figure 9 fig9:**
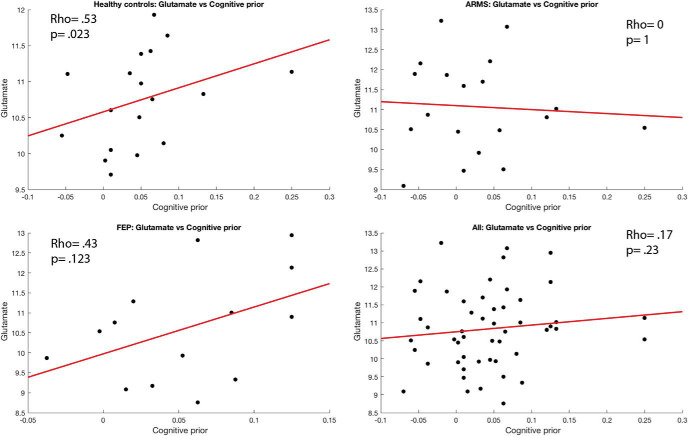
Correlations between cognitive prior strength and glutamate levels for all groups. We report Spearman’s correlations but plot linear fits for display purposes. ARMS = at-risk mental state patients; FEP = first-episode psychosis.

Correlations with glutamate were tested in a subset of participants, namely 18 healthy controls, 19 ARMS, and 14 FEP patients. We looked for a correlation across all participants between glutamate levels and the strength of the perceptual and cognitive priors but found no significant correlation (perceptual: ρ = .18, *p* = .21; cognitive: ρ = .17, *p* = .23). When exploring the correlations in the separate patient groups, we found that there was a significant positive relationship between glutamate levels and cognitive priors in the control group (ρ = .53, *p* = .023) but not with perceptual priors (ρ = .294, *p* = .24). In the ARMS group, no significant correlations were found for either cognitive (ρ = .0, *p* = 1) or perceptual priors (ρ = .07, *p* = .78). In the FEP group, a significant correlation was found with perceptual (ρ = .57, *p* = .035) but not cognitive priors (ρ = .43, *p* = .128). As these findings were secondary to the core hypothesis in the present chapter, they were not corrected for multiple comparisons. The effects did not remain significant when they were controlled for multiple comparisons (see Figures 8 and 9).[Fig-anchor fig8][Fig-anchor fig9]

#### Delusion ideation and hallucinations correlated differently with prior in different patient groups

To explore the relationship between the usage of sensory and cognitive priors and the relation with symptoms, we computed Spearmen correlations within the different experimental groups (see Table 2). In brief, we found that an increase in cognitive prior use was associated with delusion ideation in ARMS, whereas a decrease in the usage of perceptual priors was associated with perceptual abnormalities and delusion ideation in the FEP group. No significant correlations were found in the control group, or in a combined ARMS+FEP group.[Table-anchor tbl2]

## Discussion

In the present study, we found that whether prior expectations have a stronger or weaker effect on perception in psychosis depends on the origin of the prior expectation and the disease stage. We found strong evidence of weakened perceptual priors in the ARMS group compared to the FEP group and some evidence of ARMS versus controls differences. In contrast, when comparing cognitive priors, we found that the FEP group had stronger priors compared to the ARMS and healthy control group, whereas the healthy controls and ARMS group did not differ from each other.

The present findings can be interpreted in the hierarchical predictive coding framework. This framework suggests that the brain models the world by making predictions about upcoming sensory input that are subsequently updated by discrepancies between the predictions regarding the sensory input and the actual sensory input, termed the prediction error (Bastos et al., 2012; Clark, 2013; Friston, 2005; Hohwy, 2013; Knill & Pouget, 2004; Rao & Ballard, 1999). In these models, abnormal perception and delusional beliefs can be expected to occur when the balance between the prior expectations and sensory input is shifted (Fletcher & Frith, 2009), as was found in the present experiment. That is, sensory input can come to dominate perception, likely resulting in the subjective experience of being overwhelmed by their sensory environment and attributing importance to otherwise irrelevant stimuli, as is sometimes reported in the early, including prodromal, stages of psychosis (Bowers, 1968; Corlett et al., 2011; Freedman & Chapman, 1973; McGhie & Chapman, 1961).

Our results of abnormally strong high-level priors in first-episode psychosis, all of whom had either current or recent delusions, are in accordance with previous postulates (e.g., Adams et al., 2013; Sterzer et al., 2018). We further note that high-level, cognitive priors were stronger in established psychosis compared to the ARMS, consistent with previous theory that strong high-level priors may develop subsequent to weak low-level priors (Adams et al., 2013; Sterzer et al., 2018). As Heinz et al. (2019) reasoned, “reduced precision of *perceptual beliefs* encoded at low levels, e.g. in sensory cortices, may be compensated by increased precision of more abstract *conceptual beliefs* encoded in higher-level brain circuits” (emphasis in original; p. 1096). However, previous theories have not described on what time scale this compensation happens, and no previous studies have examined over what time scale or at what stages in psychotic illness this may occur. Our data suggest that this compensation may not necessarily be instant but might develop over time, possibly in the transition from the prodromal stage (ARMS) to frank psychosis (FEP).

A recent study examining the influence of prior expectations on auditory perception used a conditioning paradigm to study aberrancies in healthy voice hearers, voice hearers with psychotic illness, and psychotic illness without voice hearing (Powers et al., 2017). Individuals who heard voices were susceptible to reporting hearing a sound when none was present following a previously associated cue. Computational modeling showed that individuals with psychotic illness had difficulties learning that a cue failed to predict a sound, sticking to their prior expectations, whereas individuals who heard voices but did not have psychotic illness did recognize volatility and were able to alter high-level beliefs. This might, in part, explain why we only see an effect of the cognitive priors in the psychosis group but not in the at-risk mental state group, who, although help seeking, do not (yet) have psychotic illness. Because the current paradigm involves a staircase experiment, we only picked up strong effects of prior expectations in individuals who remained influenced by the priors toward the end of the experiment. The individuals at risk for psychosis might have been influenced in the task early on but changed their expectations regarding the validity of the cue later on. Since our key variable is the influence of the priors at convergence, we might have been unable to pick this up.

It should be noted that there are several outliers in the first-episode psychosis group. Although our statistical tests are robust against Type I errors in a data set with outliers (Zimmerman, 1994), and the results hold when removing these outliers, it still raises the question of what the nature of the outliers is. One possibility is that there is a subset of individuals that is exceptionally strongly influenced by prior expectations. Indeed, previous studies have reported nonnormal data on such variables (see Powers et al., 2017, Figure 1E). However, there is also the possibility that these participants performed the task differently or misunderstood the instructions, although we have no evidence that these outliers were caused by experimental measurement error. The reliability of the perceptual priors was slightly less in the FEP group compared to the other groups, but it should be noted that this difference was not significant, and there was still a reliable correlation between the independently assessed prior strengths (correlation 0.7 for use of sensory priors in FEP). In addition, an average was taken from the two independently assessed priors, likely increasing the reliability further. Furthermore, since the present task does not measure performance but rather a perceptual bias, an increase in noisy decision-making would not bias the results one way or the other.

It has been argued that there might be a relationship between early sensory processing deficits and high-level deficits in schizophrenia (Leitman et al., 2010). This raises the question of what the exact nature of this relationship is and whether it might be relevant in understanding the development of psychosis. While the cognitive and perceptual prior strengths were weakly (ρ = 0.24), but significantly, correlated in the sample as a whole, the strengths of the correlations were significantly different across groups. Although we acknowledge the caveat that within-group correlations were of secondary interest in this study, and not well-powered, the fact that the group comparisons in strengths of priors were sensitive to whether priors were high or low level provides supporting evidence that level of priors does matter in this research context. Perceptual priors in ARMS were negatively correlated with cognitive priors, whereas in FEP and healthy controls, and the sample as a whole, the correlation was positive. We speculate on the possibility that an increase in the influence of cognitive priors on perception in the FEP group is an adaptation to early visual processing deficits in the earlier stages of psychosis, as seen in the at-risk group. This increase in cognitive priors subsequently could potentially act to counter the decrease in diminishment of perceptual priors explaining the positive correlation that is observed in the FEP group. This increase in cognitive priors may manifest themselves as delusions on the phenomenological level, as can be seen in both the strong cognitive priors in FEP and in the correlation with symptom severity in the ARMS group. Subsequently, if perceptual priors remain low in the FEP stage, this is correlated to worse symptomology, suggesting that a failure for the brain to deal with a change in the perceptual system may be important for psychopathology severity in this stage of the illness. Interestingly, in the FEP stage, there is no correlation between cognitive priors and symptoms, possibly due to noise added to the data through the effects of treatment, recovery in some, and delusional belief formation being an attempt at making sense of a changing sensory world (Mishara & Corlett, 2009). Overall, our data emphasize the importance of distinguishing between priors at high and low levels of the cognitive hierarchy (Schmack et al., 2013).

**Figure 10 fig10:**
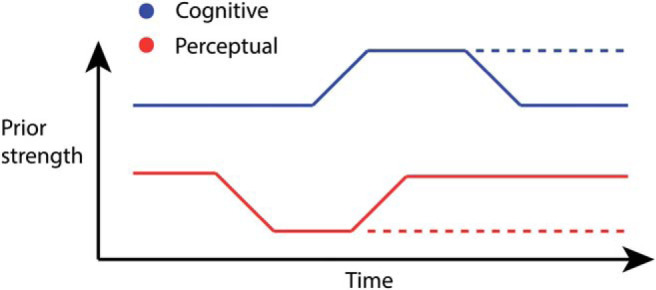
A proposed model for the interaction between different levels of prior over time in psychosis. The early stages of psychosis might be characterized by a weakening of lower-level perceptual priors as indicated by a fall in the lower red (dark gray) line. This causes a shift in the strength of cognitive priors (as indicated by an increase in the blue [light gray] line) as an attempt to explain the abnormal perceptual experiences, causing positive symptoms of psychosis. This will counter the weakening of lower-level priors. A failure to attenuate the weakening of lower-level priors may result in more severe, sustained symptoms, as indicated by the dashed lines.

We conclude that the initial stages of psychosis may be characterized by a weakening of lower-level perceptual priors. Compensatory neural systems changes may lead to deploying stronger higher-level priors in order to deal with the increased strong drive on perceptual input. These changes might be associated with formation of delusional beliefs (as supported by the correlations with symptoms). If this compensatory strategy is effective, the weakened perceptual priors may be restored throughout development. If ineffective, the perceptual priors remain weak, and psychotic symptoms maintain (as supported by the correlations with symptoms). This model is described in Figure 10, where the strengths of perceptual and cognitive priors are depicted in red (dark gray) and blue (as indicated by an increase in the blue [light gray] line), respectively, over time in psychosis, and the dotted line indicates worse clinical outcome in some patients. This model can be tested in longitudinal designs to clarify the temporal and causal relationship between the different priors.[Fig-anchor fig10]

Two previous studies have looked at the McGurk effect in schizophrenia. White et al. (2014) found that patients were, on average, less vulnerable to the illusion than controls, with a strong relationship with duration of illness, such that individuals who have been ill for longer were less likely to report a McGurk effect (White et al., 2014). Pearl et al. (2009) used a more complex recruitment design and had more mixed results that interacted in a complex fashion with age; the interpretation of their patient results are made challenging given that results in controls interacted with age in an unexpected manner. In these previous studies, participants were required to report binary choices on whether they perceived the McGurk effect, whereas we used a staircase procedure to examine the degree of influence that lip movements have on auditory perception. We did not find a diminishment in the degree that lip movements influenced auditory perception in psychosis patients. This might relate to differences in methodology or perhaps to the age difference between our study (mean age 24.9 years) and White’s study (mean age 39.0 years) given that the absence of illusory effect was more marked in White et al.’s older patients with longer disease duration. Further studies looked at the ability for schizophrenia patients to use lip movements to understand written speech, which found aberrancies in schizophrenia, while general lip-reading capabilities remained intact (de Gelder et al., 2003; Myslobodsky et al., 1992; Pearl et al., 2009; Ross et al., 2007; Szycik et al., 2013). Again, the patient groups in these studies consisted of schizophrenia patients who were older and in a more chronic phase than in the present sample, potentially explaining the discrepancy with the present study.

In the present study, we have described our effects in terms of an increase or decrease in the influence of prior expectations. However, it should be noted that the present paradigm is not able to directly discern whether a stronger influence of prior expectations in auditory perception is due to a change in the strength of the prior or a weakening in the strength of the sensory input. Indeed, previous studies have shown impairments in the ability to do simple auditory discrimination tasks in schizophrenia (Javitt & Sweet, 2015). Future studies could utilize simple auditory discrimination tasks to explore whether these effects are driven by these deficiencies or whether they can be separated.

It has been proposed that glutamatergic abnormalities may be prominent in the early stages of psychotic illness (Kumar et al., 2018; Merritt et al., 2016) and that these may be key in driving pathophysiology of illness, predictive processing dysfunction, and psychopathology (Corlett, Frith, & Fletcher, 2009; Corlett et al., 2011; Sterzer et al., 2018). We did not find a significant relationship between glutamate levels in the anterior cingulate cortex and the strength of the perceptual and cognitive priors across all participants. However, in exploratory analyses, we analyzed the groups separately, and here we did find that in the healthy group, there was a significant positive relationship between anterior cingulate glutamate levels and cognitive priors, and in the FEP group, there was a significant relationship between glutamate levels and perceptual priors. This relationship between anterior cingulate glutamate levels and perceptual priors in the FEP group is interesting as the correlations suggest that a (sustained) weakening of perceptual priors is particularly relevant to FEP symptomology; thus, glutamate might play a role in having sustained weakened perceptual priors. The absence of a correlation with the cognitive priors might be due to a lack of power as a successful glutamate scan was only acquired from 14 individuals who had first-episode psychosis. We report MRS results uncorrected for multiple comparisons, which should currently be viewed as preliminary. Larger sample size studies on glutamate levels, the strength of perceptual priors in psychosis, and their interrelation will be required to confirm (or refute) our results, which should currently be interpreted with caution. A further limitation of our MRS work is the use of a single region, located in the anterior cingulate cortex, from which our glutamate measure was drawn. We do not mean to imply that this is the only region influencing the role of priors in decisions, but until MRS technology matures to allow simultaneous acquisition of neurochemistry measures across the whole brain, a priori region of interest selection will remain the norm.

As with all studies that use the at-risk mental state construct, there is an inherent limitation in terms of the inability to prospectively determine whether an at-risk individual will develop a first episode of psychosis. Therefore, future studies would benefit from following up with individuals determined to be in the at-risk group and exploring the predictive validity of a change in the usage of priors. Indeed, longitudinal studies will be required for definitive conclusions about how use of priors relates to illness stage.

Extending this research beyond the field of psychosis, we note that autism has been suggested to also be associated with a weakening of priors, but it usually does not develop into psychotic symptoms (Lawson, Rees, & Friston, 2014; Pellicano & Burr, 2012; van Boxtel & Lu, 2013), although there are increased rates of psychotic symptoms in autism and other neurodevelopmental disorders (Hussain & Murray, 2015; Larson et al., 2017). The difference between schizophrenia spectrum psychosis and autism may lie in the fact that autism presents itself in early childhood, whereas schizophrenia spectrum illness typically develops later in adolescence. The consequence of this is that during the emergence of schizophrenia spectrum psychosis, the brain has to explain a changing world, whereas the sensory-driven world of autism presents itself at birth, requiring no changes in the model of the world to form (i.e., no formation of delusional beliefs), yet the experience of being overwhelmed by sensory experiences remains. Future experiments would need to use a longitudinal approach to support this hypothesis, namely that psychosis is preceded by a decrease in the influence of perceptual priors on perception, followed by a normalization accompanied by an increase in higher-level cognitive priors, whereas autism has weakened priors from birth. In order to test such hypotheses, longitudinal paradigms are preferred, which require potentially large groups of people. In order to acquire such amounts of data, the possibility of online testing could be considered, to which the present experiments are well-adapted due to the simplicity of the paradigm and the brief duration of the experiments (10 min each).

We acknowledge the complexity of experimentally manipulating “cognitive” as opposed to “perceptual” priors, and we caution against simplistic interpretations of the results of this and previous studies that attempt to address predictive coding at different hierarchical levels. It can be challenging to relate the hierarchical level of differing cognitive processes; a process considered by one group of researchers as “cognitive” could be considered to be “perceptual” by others. We note the limitation that the sensory and cognitive priors in our study were not explicitly computationally modeled as different levels in a cognitive/cortical hierarchy.

While acknowledging these limitations, we contend that our study provides initial evidence that the influence of perceptual priors might be weakened in the early stages of psychosis but not in the later stages, whereas cognitive priors may be strengthened in the later stages but not early stages. Therefore, we suggest that previously reported inconsistencies in the literature regarding the influence of prior expectations on sensory processing might be due to differences in the origin of the prior expectation and the disease stage. Furthermore, changes in perceptual and cognitive priors might interact with each other throughout the development of psychosis; cortical glutamate might play a mediating role in the process.

## Figures and Tables

**Table 1 tbl1:** Participant Demographics and Symptom Scores

Measure	HCS *n* = 32	ARMS *n* = 29	FEP *n* = 30	*p*
PANSS	13.1 (4.6)	26.7 (12.1)	31.6 (12.3)	<.001
Positive	6.5 (2.3)	13.6 (5.7)	18.0 (6.9)	<.001
Negative	6.6 (2.4)	13.1 (7.5)	13.6 (7.7)	<.001
MFQ	8.5 (5.1)	33.2 (17.4)	31.8 (23.6)	<.001
CAPS	32.9 (1.4)	44.1 (7.0)	43.6 (9.7)	<.001
Distress	1.6 (3.0)	29.8 (20.9)	32.1 (33.9)	<.001
Intrusive	2.2 (3.7)	34.9 (22.8)	38.5 (37.4)	<.001
Frequency	1.3 (2.3)	28.3 (17.8)	29.7 (31.1)	<.001
PDI total	22.4 (1.5)	29.3 (4.5)	31.1 (6.5)	<.001
Distress	2.4 (2.8)	24.1 (16.9)	28.0 (23.9)	<.001
Intrusive	2.4 (2.7)	23.6 (17.4)	29.5 (22.9)	<.001
Conviction	3.6 (4.0)	24.9 (15.9)	31.0 (25.3)	<.001
Age	22.4 (3.7)	21.8 (3.5)	25.1 (4.8)	<.01
*N* male	17	21	24	>.05
*Note*. HCS = healthy control sample; ARMS = at-risk mental state patients; FEP = first-episode psychosis; PANSS = Positive and Negative Symptoms Scale; MFQ = Mood and Feelings Questionnaire; CAPS = Cardiff Abnormal Perceptions Scale; PDI = Peters Delusion Index Scale. The table values indicate means and standard deviations in parentheses or *M* (*SD*).				

**Table 2 tbl2:** Correlations Between Abnormal Perception and Belief and Usage of Sensory and Cognitive Priors Across All Groups

Questionnaires	ARMS		FEP		ARMS + FEP		HCS	
	Sensory	Cognitive	Sensory	Cognitive	Sensory	Cognitive	Sensory	Cognitive
PDI								
*p*	.44	.030	.023	.19	.09	.28	.92	.27
ρ	−.16	.44	−.48	−.29	−.25	−.16	.018	.21
CAPS								
*p*	.84	.87	.008	.16	.10	.86	.06	.56
ρ	.044	.037	−.55	−.31	−.24	.03	.34	.11
*Note*. ARMS = at-risk mental state patients; FEP = first-episode psychosis; HCS = healthy control sample; PDI = Peters Delusion Index Scale; CAPS = Cardiff Abnormal Perceptions Scale.
